# Single institution followed by national implementation of systematic surgical quality control and feedback for radical prostatectomy: a 20-year journey

**DOI:** 10.1007/s00345-019-02887-4

**Published:** 2019-08-06

**Authors:** J. Stranne, E. Axen, I. Franck-Lissbrant, P. Fransson, M. Frånlund, J. Hugosson, A. Khatami, K. Koss-Modig, P. Lodding, M. Nyberg, P. Stattin, O. Bratt

**Affiliations:** 1 Department of Urology, Institute of Clinical Science, Sahlgrenska Academy, University of Gothenburg, Gothenburg, Sweden; 2 Department of Urology, Sahlgrenska University Hospital, Region Västra Götaland, Gothenburg, Sweden; 3 Department of Oncology, Institute of Clinical Science, Sahlgrenska Academy, University of Gothenburg, Gothenburg, Sweden; 4 Department of Oncology, Sahlgrenska University Hospital, Region Västra Götaland, Gothenburg, Sweden; 5 Department of Nursing, Umeå University, Umeå, Sweden; 6 Department of Surgical Sciences, Uppsala University Hospital, Uppsala, Sweden

**Keywords:** Prostate cancer, Radical prostatectomy, Quality control, PROM

## Abstract

**Purpose:**

The demand for objective and outcome-based facts about surgical results after radical prostatectomy (RP) is increasing. Systematic feedback is also essential for each surgeon to improve his/her performance.

**Methods:**

RP outcome data (e.g., pT-stage and margin status) have been registered at Sahlgrenska University Hospital (SUH) since 1988 and patient-related outcome measures (PROM) have been registered since 2001. The National Prostate Cancer Registry (NPCR) has covered all Regions in Sweden since 1998 and includes PROM-data from 2008. Initially PROM was on-paper questionnaires but due since 2018 all PROMs are collected electronically. In 2014 an on-line “dashboard” panel was introduced, showing the results for ten quality-control variables in real-time. Since 2017 all RP data on hospital, regional, and national levels are publicly accessible on-line on “www.npcr.se/RATTEN”.

**Results:**

The early PROM-data from SUH have been used for internal quality control. As national clinical and PROM-data from the NPCR have been made accessible on-line and in real-time we have incorporated this into our pre-existing protocol. Our data are now internally available as real-time NPCR reports on the individual surgeons’ results, as well as ePROM data. We can compare the results of each surgeon internally and to other departments’ aggregated data. The public can access data and compare hospital level data on “RATTEN”.

**Conclusions:**

The process of quality control of RP locally at SUH, and nationally through the NPCR, has been long but fruitful. The online design, with direct real-time feedback to the institutions that report the data, is essential.

## Introduction

The methods for quality control of surgical procedures, such as radical prostatectomy, have dramatically changed over time. When Hugh H. Young described the radical prostatectomy procedure in four cases in 1905 [1], surgeons provided their own quality control, with no external validation. After the anatomic radical prostatectomy was described by Walsh and co-workers in the early 1980s [2], the number of prostatectomies soared and the need for systematic quality control became evident [3]. The principal goals of the procedure remain the same since Walsh’s pioneering work, i.e., cancer control, urinary continence and preserved erectile function—“trifecta”. However, patients and health care providers have often had to rely on the individual surgeons’ self-claimed excellent results and results published by high-volume tertiary centres. Today, in the information era, and with increasing surgical volumes, this is clearly insufficient. Both patients and health care providers demand objective and outcome-based facts about surgical results. Moreover, for the individual surgeon, detailed feedback on their results to raise their self-knowledge from subjective high self-confidence to objective individual results regarding both cancer control and patient-reported outcomes measures (PROM) such as continence, erectile function and mental well-being is essential.

This paper describes the journey from local initiatives to a standardised nationwide quality-control system for radical prostatectomy patients in Sweden. This now enables detailed information for patients, public health care providers, decision makers and individual surgeons, and allows for benchmarking of both patient selection and the execution of this common surgical procedure.

## Materials and methods

In 1987 the first regional prostate cancer registry started in the south-western region of Sweden. During the 1990s the number of regional registers of various aspects of prostate cancer care increased successively and these have been merged to the National Prostate Cancer Registry (NPCR). At Sahlgrenska University Hospital in Gothenburg, radical prostatectomy outcome data have been prospectively collected since 1988. PROM was added in January 2001. A baseline questionnaire was given to patients before surgery that included questions on continence, erectile function and inguinal hernia formation. Follow-up questionnaires were sent by mail at 3, 6, 12, 18, 24, and 36 months after surgery. Feedback from PROM-data was given to each individual surgeon regularly and were openly discussed within the group.

In 2004 the preparations started for the first national prostate cancer guidelines. The guideline group saw the need for a national PROM questionnaire to enable comparisons between hospitals, but it took several years before agreement was reached on which questions to use. In 2008 the National Prostate Cancer Register (NPCR) of Sweden expanded the number of variables registered and incorporated the first version of a national PROM questionnaire for the assessment of side-effects and symptoms after curative treatment with either radiotherapy or surgery for non-metastatic PC [4]. The paper-based questionnaire was handed out to patients at the treating unit together with a stamped and addressed return envelope, in which to send it to the Regional Cancer Centre (North) in Umeå, where the data were optically scanned and stored. Follow-up questionnaires were subsequently sent directly to patients at 6 and 18 months and at 3 and 5 years after treatment.

Only a few centres, mainly those that had a pre-existing routine for collecting PROM, obtained satisfactory response rates on this paper-based questionnaire; for instance, at Sahlgrenska University Hospital in Gothenburg a nurse kept record of the questionnaires and reminded patients over telephone to fill them in. Moreover, scanning questionnaire responses and data management was time consuming so the data were presented a very long time after surgery to the surgeons. Due to the low capture rate and the labour-intense logistics to merge data in these paper forms with data in NPCR, on-line questionnaires (ePROM) were launched in 2018. Sahlgrenska University Hospital piloted ePROM and subsequently all departments that treat prostate cancer patients in Sweden were offered to use the ePROM. Patients can now answer the ePROM on-line at the outpatient ward or at home and the results are accessible in real-time. An English version of the ePROM questionnaire is attached as an appendix [5]. After treatment, letters are sent at 3, and 12, and 36 months with a code to an on-line questionnaire. A reminder is automatically sent after 4 weeks to men who have not responded.

In 2015 a specific radical prostatectomy register was introduced. The surgeons fill in an on-line form immediately after the procedure. The surgeons’ identity is coded and the code key is kept at the department. Later, other staff report post-operative blood transfusion, any complications during the procedure and the final histology.

All units have on-line access to all of their own data and can compare the results for the individual surgeons at the clinic, and can also compare their results with other units’ aggregated results and the national average. All data in NPCR can be presented for specific time periods and for specific groups of patients, defined by age, cancer characteristics, base-line erectile function, et cetera.

**Fig. 1:**
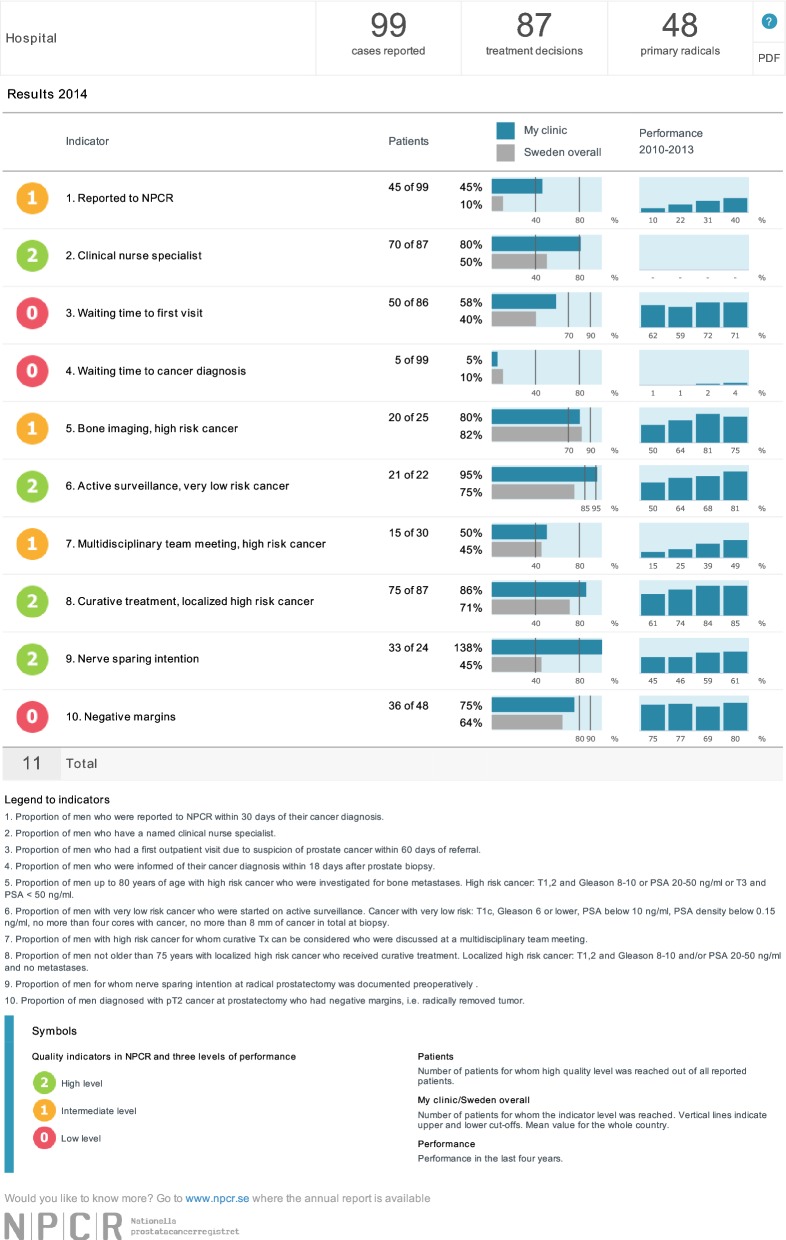
Screenshot from the National Prostate Cancer Registry’s (NPCR’s) on-line real-time “dashboard” showing the 10 selected quality control variables. The variables are chosen to represent pertinent aspects of the care provided and the level of performance for each indicator, based on aims set by the national steering board of the NPCR, is coded green (high level), yellow (intermediate level) or red (low level), to give an immediate overview of the unit’s performance

An on-line “dashboard” panel was introduced in 2014, which in real time shows the results for ten selected quality-control variables for the past 12 months [6]. The variables are chosen to represent pertinent aspects of the care provided and the level of performance for each indicator, based on aims set by the national steering board of the NPCR, is coded green (high level), yellow (intermediate level) or red (low level), to give an immediate overview of the unit’s performance (Fig. 1). A similar dashboard for oncological prostate cancer services was introduced in 2015. The proportion of nerve-sparing procedures for low- and intermediate-risk tumours and the proportion of negative surgical margins for pT2 tumours are selected as important quality indicators and included on the urological “dashboard”. As both overtreatment of low-risk prostate cancer and undertreatment of localised high-risk prostate cancer, the latter especially in elderly men, remain a problem, indicators of these parameters are also included in the dashboard [6, 7]. Once a parameter reaches a green, high level, of performance it can be changed by the national steering board to a different indicator where the level of performance on a national level is insufficient.

**Fig. 2:**
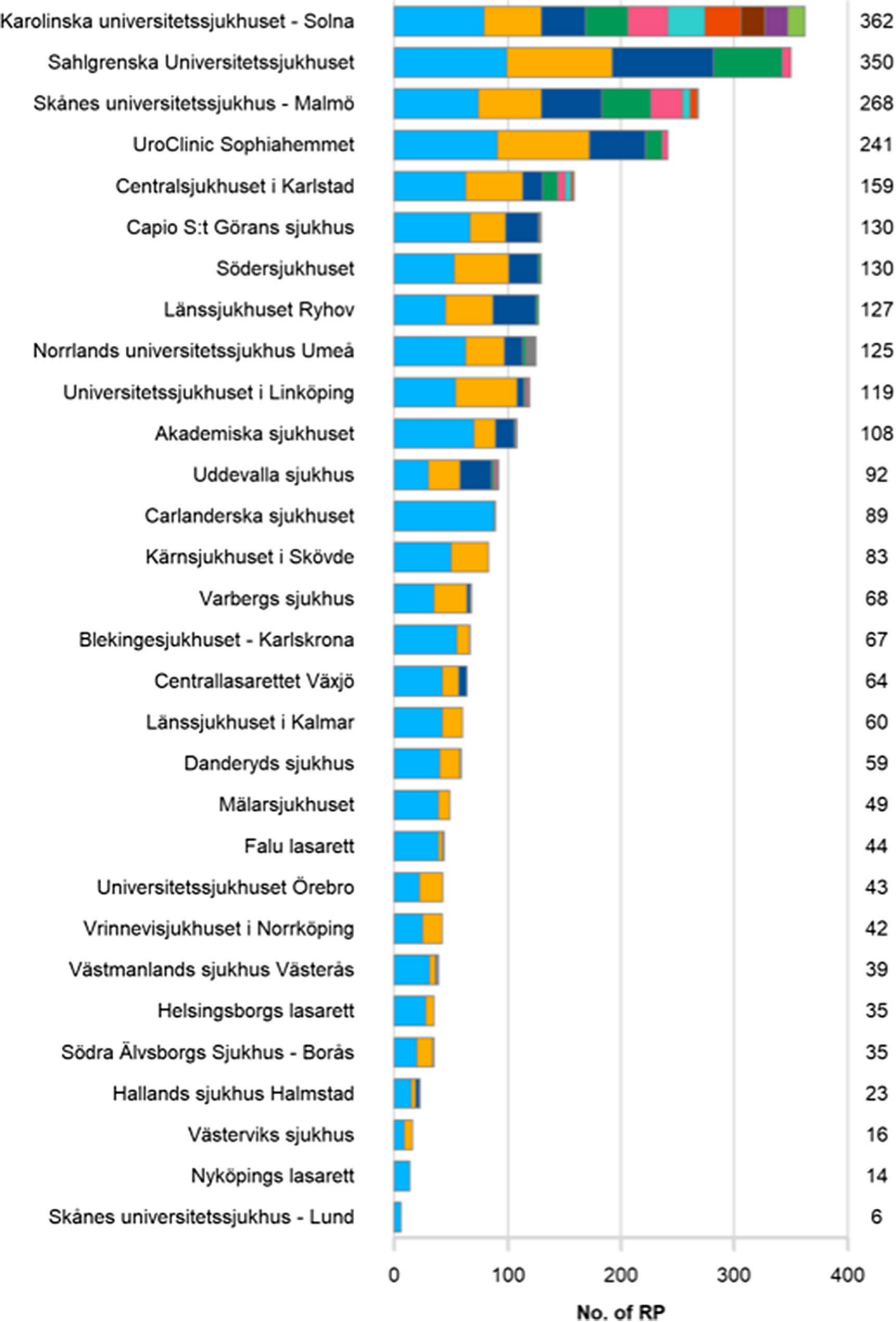
Screenshot from “RATTEN”. Number of radical prostatectomies per surgeon per hospital in 2018. Each colour in the hospitals’ bar represents one surgeon

**Fig. 3:**
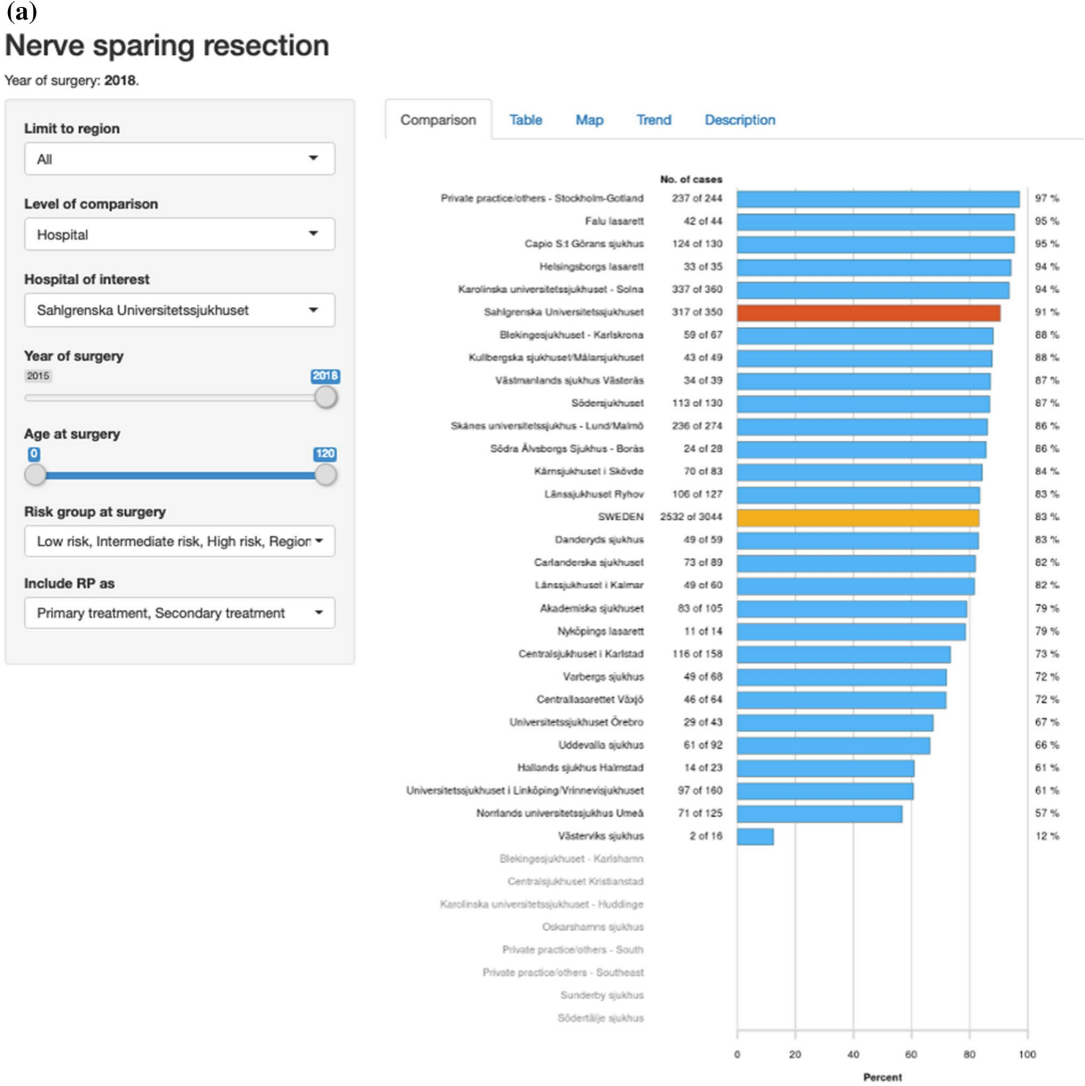

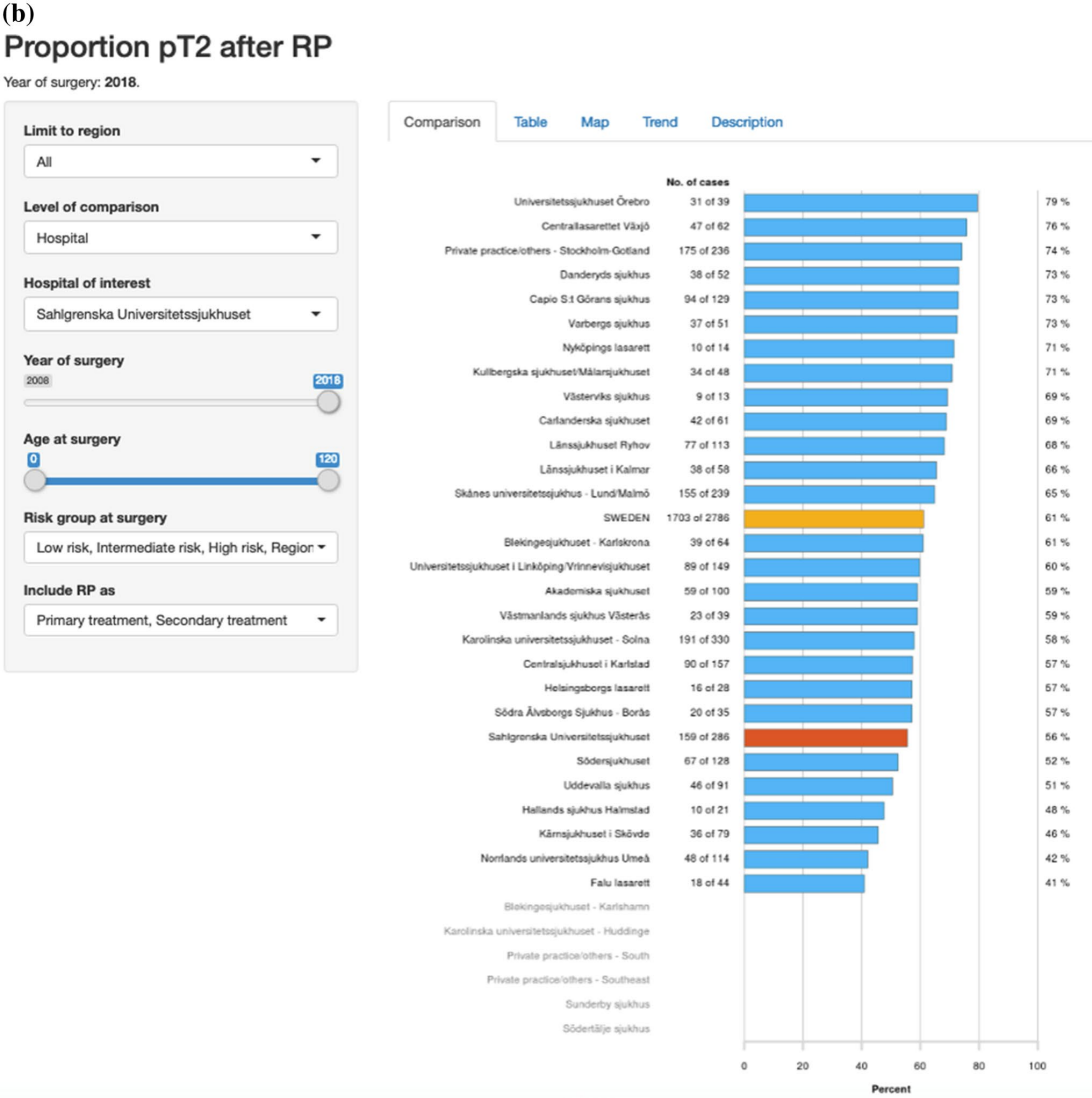

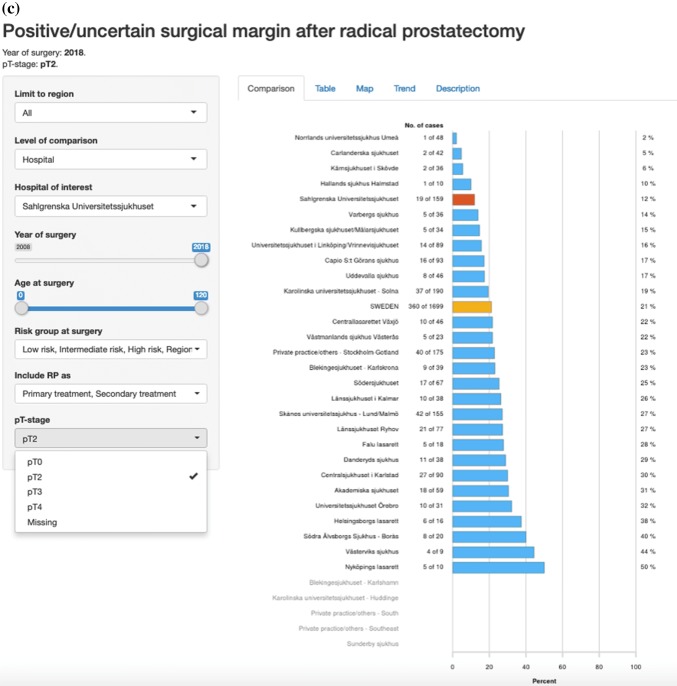

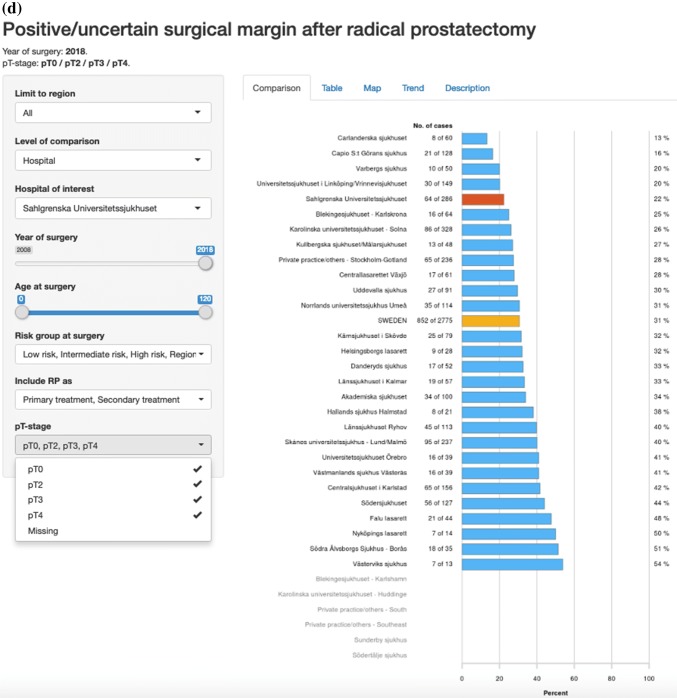
Screenshots from “RATTEN”. All risk groups are included. The Swedish national average is shown as a yellow bar and Sahlgrenska University Hospital is selected as the hospital of interest and is therefore shown as a red bar. Proportions of nerve-sparing procedures per hospital (**a**), proportion of pT2 tumours (**b**), rate of negative surgical margins in pT2 specimens (**c**) and rate of negative surgical margins for all pT stages (**d**)

In 2017 another step forward was taken when NPCR made data publicly available on-line (www.npcr.se/RATTEN) [8]. On this web-site radical prostatectomy data are now publicly available on national, regional and local levels for a given calendar year or merged for several years: The number and type (open, robotic) of procedures, the number of surgeons and how many prostatectomies each surgeon performed (Fig. 2), the number and proportion of nerve-sparing procedures and of pT2 tumours, and the number and proportion of specimens with cancer in the surgical margin, stratified by pT-stage (Fig. 3). Furthermore, data on various waiting times (e.g., from referral to diagnosis, from diagnosis to treatment), on diagnostics (e.g., distribution of risk classifications, number of needle biopsies) and on choice of primary treatment (e.g., active surveillance, surgery, radiotherapy or watchful waiting) are also published. All these data can be stratified according to cancer risk group. Individual surgeon’s results are accessible only at their own department.

Parallel to the development of the national register data, Sahlgrenska University Hospital’s efforts to secure quality control of radiology, pathology, and short-term complications/re-admittance rates have continued. Every second week there is a prostate MRI conference, where all scans for the next 2 weeks’ cases are demonstrated and the surgical plan is discussed. All procedures are video recorded and saved in a shared data base, so that all surgeons can view each other’s procedures and discuss complications and technical issues. Every other second week the surgeons meet with a pathologist who demonstrates all specimens with a pT3–4 cancer and/or a positive surgical margin. The degree of nerve sparing and other technical issues are discussed in the light of the preoperative information and the postoperative findings. As a complement to the NPCR a local re-admission registry is kept of all patients undergoing surgery at Sahlgrenska University Hospital. A local “re-admittance record” of all procedures performed at the department is also kept to identify any deviancy from the expected post-operative course.

**Fig. 4:**
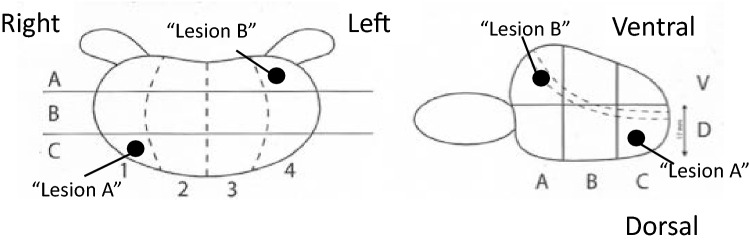
Common template of the prostate used by urologists, pathologists and radiologists throughout Sweden. The location of a lesion is described by coordinates starting from right to left by a number (1–4), followed from base to apex by a letter (A–C) and finally dorsal (D) or ventral (V). The location of “Lesion A” in the figure would be described as 1CD and “Lesion B” as 4AV

A spin-off effect of the work with quality indicators, which also included pathologists and radiologists, was a mutual understanding of the need for standardising anatomic definitions. Based on a consensus between the pathology, radiology, and urology departments at Sahlgrenska University Hospital, a national template of the prostate was established in 2017 and is recommended in the national guidelines for prostate cancer care (Fig. 4).

## Results

The early PROM-data from Sahlgrenska University Hospital have been used for internal quality control as well as identification of new potential complications from the start. In 2006 we could identify a 20% rate of inguinal hernia within 2 years after open radical prostatectomy [9], which was not a recognised complication at that time. In 2014 we analysed the inter-individual surgeon variability of oncologic and functional results and identified a greater heterogeneity within the group of surgeons than expected [10]. Initially, the internal reports were somewhat unstructured, but in 2015 a systematized feedback was introduced, with regular quarterly reports showing all individual surgeon’s results for pT-stratified margin status and 3- and 12-month continence and erectile function.

**Fig. 5:**
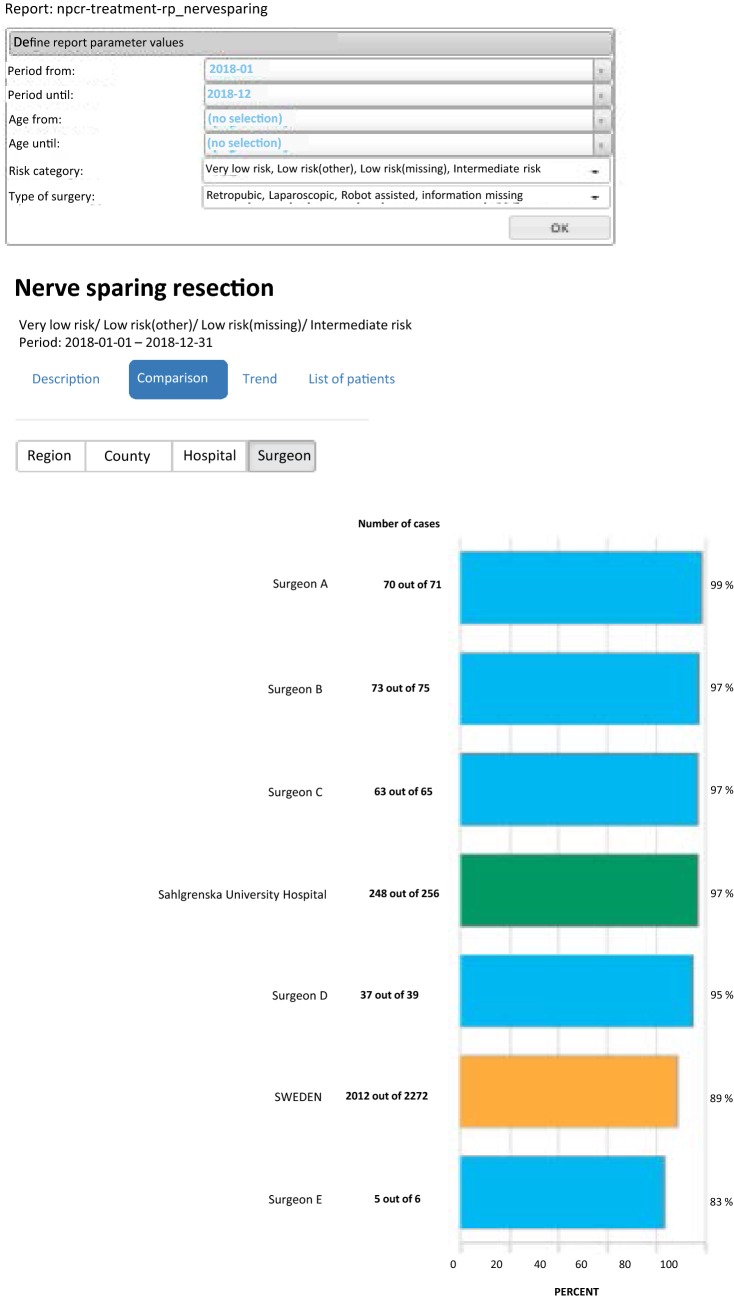

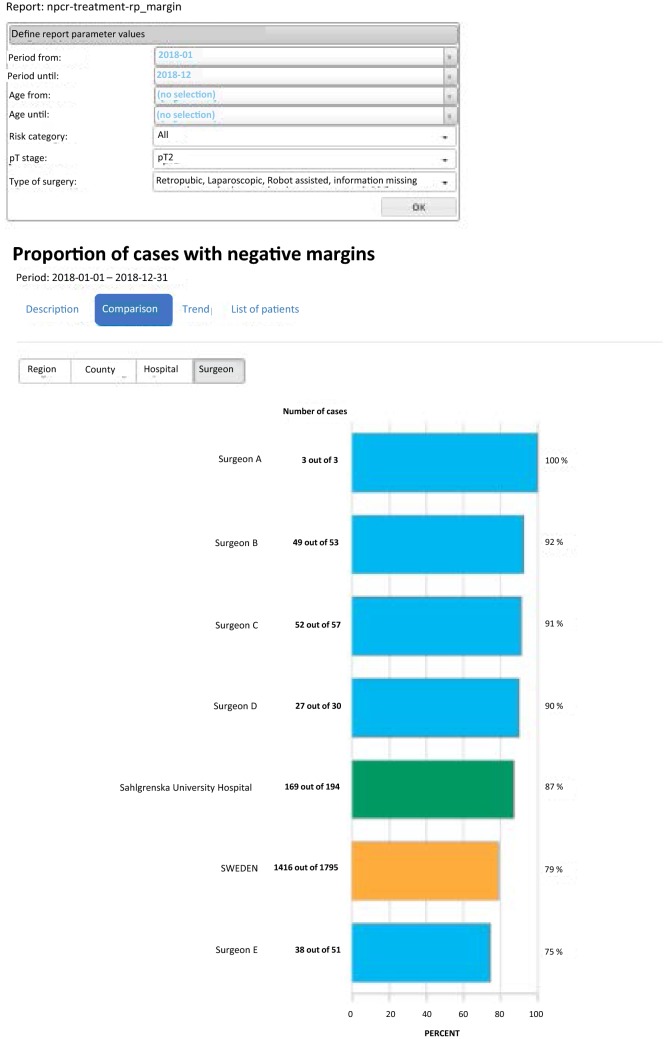
Screenshots from the National Prostate Cancer Registry’s (NPCR’s) on-line real-time reports showing the proportion of nerve-sparing procedures for low- and intermediate-risk cancers per surgeon at Sahlgrenska University Hospital (**a**) and the proportion of negative surgical margins for pT2 tumours per surgeon at Sahlgrenska University Hospital (**b**)

**Fig. 6:**
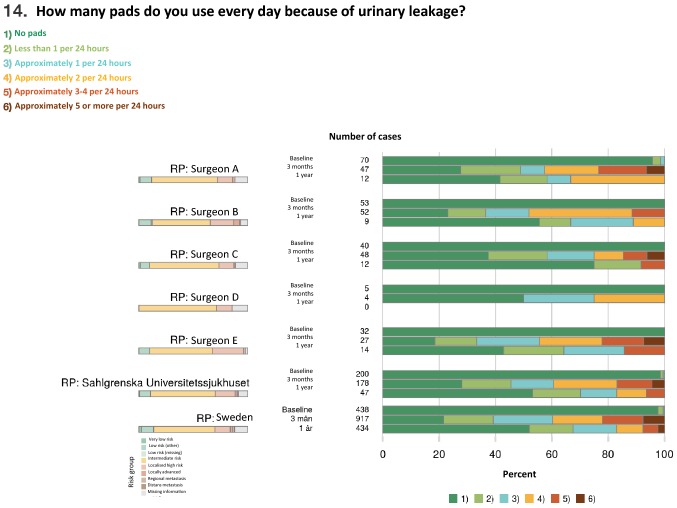
Screenshot from National Prostate Cancer Registry’s (NPCR’s) on-line real-time report showing daily pad use for urinary leakage before and 3 and 12 months after surgery per surgeon at Sahlgrenska University Hospital compared with the hospital and national average. The colours of the bars to the left show the risk-group distribution and of the bars to the right the continence, ranging from “No pad” (dark green) to “Around 5 or more pads per 24 h” (brown). All data in the report are from the ePROM questionnaire introduced in January 2018, hence the low number of patients who completed the 12 months questionnaire

The overall coverage of the NPCR has been very high in most clinics from the start in 2008 and is today as high as 98% [4]. Over the years we have incorporated the NPCR and ePROM into our pre-existing quality control protocol, and phased out the local paper-based PROM. The coverage of paper-based PROM has been relatively stable between 70 and 80% and this level has been maintained after the introduction of the ePROM. A work to identify which patients do not answer the ePROM and the reasons behind this is ongoing with the aim to increase the level of coverage to as near 100% as possible. Our data are now internally available as real-time NPCR reports on the individual surgeons’ results, such as the proportions of nerve sparing for low- and intermediate-risk cancers and of positive margins (Fig. 5), as well as ePROM data for baseline, 3- and 12-months’ continence and erectile function (Fig. 6). We can compare the results of each surgeon within the department and the department’s aggregated data to other departments. The departments thus have access to more granulated and up-dated results than what is publicly available on RATTEN [8]. The publication of the data on this publicly available on-line platform has made comparison possible of results from different clinics by regional authorities, patient organisations and the general public. Some of the patient organisations are now organising seminars for their members on how to access the data to put pressure on the local clinic and health care providers to optimize their performance.

By use of the Sahlgrenska University Hospital’ re-admission register, we identified an increased rate of readmissions within 30 days of surgery from 6.5% in 2014 to 10.3% in 2016. An investigation group found that the increase in readmissions was mainly due to anastomotic leaks. The group reviewed the procedures videos together with the surgeons and identified some surgical steps for dissecting the apex and the urethra and suturing of the anastomosis that might have been the cause of the leaks. After a literature review a consensus decision was made by all surgeons to change the technique, which immediately led to a drop of the readmission rate to under 5% where it has remained.

After the introduction of the common prostate template in the National Guidelines for prostate cancer in 2017 it has been successively incorporated at urology, pathology and radiology departments throughout Sweden. The rate of implementation has varied between departments, hospitals and regions throughout Sweden but we are now rapidly approaching a full incorporation in clinical use by most Swedish public prostate cancer care providers including surgeons, pathologists, and radiologists.

Data from NPCR have also been used as a base for concentration of prostate cancer surgery in the western region of Sweden. There are nine publicly funded hospitals in the region and historically eight of these performed radical prostatectomies. In 2010 a total of 475 publicly funded radical prostatectomies were performed in the region. The regional health care authorities then decided to concentrate the procedures to four hospitals to ensure an annual number of procedures of at least 50–100/centre. In 2018 these four hospitals performed a total of 563 radical prostatectomies.

## Discussion

Many men have permanent side effects from prostate cancer surgery without benefitting from the procedure in terms of a prolonged life [11–13]. By excising peri-prostatic tissue widely we can increase the chance of removing the whole tumour and achieve negative surgical margins, but at the same time increase the risk of urinary and sexual dysfunction. Vice versa, sparing peri-prostatic tissue to maintain functional outcomes may result in a positive surgical margin. The provision of structured and detailed information about patient outcomes to the operating surgeons is likely to facilitate an optimal balance in the surgical planning and execution [14, 15]. However, issues related to the treatment choice have been shown to be as important to the patient as the results of surgery [16]. It is therefore of utmost importance to properly select and implement adequate quality control indicators that not only include the objective clinical outcomes of surgery, but also waiting times, staging investigations, treatment information and access to cancer specialist nurses—and how they affect the patients, using patient-reported experience and outcome measures. These indicators need to be used by each surgical centre for local quality control and improvements on surgeon and department levels. To achieve this, the reports must be easily available without delay and fed back to the surgeons with relevant benchmarking comparisons. Furthermore, the use of the same instrument for quality control across institutions is obviously also important and in the past few years there have been initiatives for developing internationally uniform PROM questionnaires. The International Consortium for Health Outcome Measures (ICHOM) has suggested a standardized set of patient-related health outcomes for tracking, comparing, and improving treatment value for localized and advanced prostate cancer [17, 18]. In the footsteps of these publications the “TrueNTH Global Registry” was established as an international registry monitoring care provided to men with localized prostate cancer, based on the ICHOM data fields and consisting of data from 25 different collecting centres, representing 113 sites in 13 countries [19]. A problem with this registry has been the differences in various variables between the local databases as well as with the ICHOM definitions. Variables such as treatment start dates correlated well and therefore had good coverage, whereas the definitions of complications and comorbidities had poor agreement between centres and thereby had low coverage in the new registry [19]. After the publication of these suggested ICHOM variables by Martin et al. and Morgans et al. an internal comparison to the NPCR variables were made [17, 18]. The NPCR steering group concluded that many of the new variables suggested by ICHOM agreed poorly with the variables that were used in Sweden since several years. The NPCR therefore decided not to change the currently used variables, as this would have made longitudinal national comparisons difficult.

Most existing PROM questionnaires used by ICHOM and others for follow-up after radical surgery for prostate cancer were recently reviewed by Protopapa et al., who concluded that the evidence is poor for that they are appropriate for assessing the outcome of individual patients in other domains than urinary and/or sexual function [20]. Hence, there is no evidence for that the data quality would be improved by changing currently used questionnaires. There is definitely a need for continuous validation and updating of the different existing questionnaires. As more and more PROM variables are being validated, a successive change towards evidence based and commonly shared variables is likely.

At Sahlgrenska University Hospital, the open and direct feedback of all prostate surgeons’ results on positive surgical margins and functional PROM data have formed the base for a discussion within the group of surgeons how to improve the results and minimize the heterogeneity within the group with the aim to reduce the previously reported heterogeneous results for the individual surgeons [10].

In Sweden, benchmarking between institutions has previously not been possible because of the low response rates of PROM. We hope that the recently launched nation-wide ePROM will increase the response rate. The demand from patient organisations, politicians, health care authorities and the public to get data to compare different care providers will be a strong incentive to take part of the national quality control system.

“Third party assessment”, i.e., independent staff assessing the outcome (not the operating surgeon) is essential to get unbiased data reported by the patients [21]. It is likely that the patients will be more truthful in their feedback if they do not directly hand over the evaluation to the person who performed the procedure. This has also been confirmed in cooperation and discussions with the patient’s representatives that have been part of the PROM-process for many years. Therefore, the follow-up questionnaires are signed by a common regional representative in each of the six Swedish health care regions.

The national consensus to make aggregated and quality-controlled data on hospital level data publicly accessible has been well received by the profession, the patient organisations and the media. Data from “RATTEN” are now also staring to be used by media as well as patient organisations to put pressure on the health care authorities and the individual clinics to optimize the results of prostate cancer surgery in Sweden. Whether or not to also publicly publish individual surgeons’ results is debated both in Sweden and internationally [22]. In the UK, each prostate cancer surgeon reports his/her results in a national, public database [23]. Arguments against open reporting include that it may lead to an aversion to take on more difficult cases, as these may “worsen” the surgeon’s results, leading to a shift in case mix towards low-risk tumours where curative treatment is unlikely to be beneficial. It could also be debatable if the surgeon him/herself should be doing the reporting, as in the UK example. A way to avoid bias and case mix shift would be the patient-reported outcomes through a “third party assessment” as in the NPCR and to include indicators such as “proportion of low risk cancer managed with active surveillance” in the quality registry to ensure that the “wrong” patients are not selected for surgery. Making individual surgeons’ results available within the department is less controversial and considered crucial for quality improvement over time [14, 15].

The nationally accepted common prostate anatomy template rendering a common language used by the radiologist, the urologist and the pathologist alike to describe the exact location of a lesion for cognitive targeted biopsies, for the planning of surgery and for comparison of confirmed lesions on preoperative imaging and postoperative histopathology. This paves the ground for further quality improvement of future diagnostics and results. However, it is too soon to draw any firm conclusions on the effect of this.

The radical prostatectomy quality control journey in Sweden is far from finished or perfect, but it is our firm belief that it is on the right track. The process of building the NPCR, and achieving its high compliance rates has been long, but the momentum has always come from within: the profession’s own drive for improvement. The online design, with real-time feedback to the reporting units, has provided an obvious benefit. Cooperating with the patients’ organisations in this process, has been extremely valuable and should not be omitted in any such work.

## Conclusions

The recipe for success in quality assurance is to choose adequate indicators, to present data in a reader-friendly manner actionable format, to dedicate enough time and resources to report data and to regularly analyse and discuss outcomes. If all these steps in the process are not well-executed, the gathering of the data is a waste of time, and surgeons will continue to believe that their results are better than they actually are.

Local and national efforts in Sweden to aggregate and feedback clinical and PROM data for the quality control of prostate cancer surgery have been successful. We argue that this has improved, and will continue to improve, the prostate cancer care for Swedish men.

## References

[1] Young HH (1905). The early diagnosis and radical cure of carcinoma of the prostate. Being a study of 40 cases and presentation of a radical operation which was carried out in four cases. Bull Johns Hopkins Hosp XVI.

[2] Walsh PC, Lepor H, Eggleston JC (1983). Radical prostatectomy with preservation of sexual function: anatomical and pathological considerations. Prostate.

[3] Walsh PC (2000). Radical prostatectomy for localized prostate cancer provides durable cancer control with excellent quality of life: a structured debate. J Urol.

[4] Van Hemelrijck M, Wigertz A, Sandin F, Garmo H, Hellstrom K, Fransson P, Widmark A, Lambe M, Adolfsson J, Varenhorst E, Johansson JE, Stattin P, Npcr Sweden PC (2013). Cohort profile: the National Prostate Cancer Register of Sweden and Prostate Cancer data Base Sweden 2.0. Int J Epidemiol.

[5] Stattin P (2019) NPCR in english. Nationella Prostatacancerregistret (NPCR). http://npcr.se/in-english/. Accessed 27 Mar 2019

[6] Stattin P, Sandin F, Sandback T, Damber JE, Franck Lissbrant I, Robinson D, Bratt O, Lambe M (2016). Dashboard report on performance on select quality indicators to cancer care providers. Scand J Urol.

[7] Cazzaniga W, Ventimiglia E, Alfano M, Robinson D, Lissbrant IF, Carlsson S, Styrke J, Montorsi F, Salonia A, Stattin P (2019). Mini review on the use of clinical cancer registers for prostate cancer: The National Prostate Cancer Register (NPCR) of Sweden. Front Med.

[8] Stattin P, Sandin F, Loeb S, Robinson D, Lissbrant IF, Lambe M (2018). Public online reporting from a nationwide population-based clinical prostate cancer register. BJU Int.

[9] Stranne J, Hugosson J, Lodding P (2006). Post-radical retropubic prostatectomy inguinal hernia: an analysis of risk factors with special reference to preoperative inguinal hernia morbidity and pelvic lymph node dissection. J Urol.

[10] Carlsson S, Berglund A, Sjoberg D, Khatami A, Stranne J, Bergdahl S, Lodding P, Aus G, Vickers A, Hugosson J (2014). Effects of surgeon variability on oncologic and functional outcomes in a population-based setting. BMC Urol.

[11] Bill-Axelson A, Holmberg L, Garmo H, Taari K, Busch C, Nordling S, Haggman M, Andersson SO, Andren O, Steineck G, Adami HO, Johansson JE (2018). Radical prostatectomy or watchful waiting in prostate cancer—29-year follow-up. N Engl J Med.

[12] Wilt TJ, Jones KM, Barry MJ, Andriole GL, Culkin D, Wheeler T, Aronson WJ, Brawer MK (2017). Follow-up of prostatectomy versus observation for early prostate cancer. N Engl J Med.

[13] Hamdy FC, Donovan JL, Lane JA, Mason M, Metcalfe C, Holding P, Davis M, Peters TJ, Turner EL, Martin RM, Oxley J, Robinson M, Staffurth J, Walsh E, Bollina P, Catto J, Doble A, Doherty A, Gillatt D, Kockelbergh R, Kynaston H, Paul A, Powell P, Prescott S, Rosario DJ, Rowe E, Neal DE (2016). 10-year outcomes after monitoring, surgery, or radiotherapy for localized prostate cancer. N Engl J Med.

[14] Breau RH, Kumar RM, Lavallee LT, Cagiannos I, Morash C, Horrigan M, Cnossen S, Mallick R, Stacey D, Fung-Kee-Fung M, Morash R, Smylie J, Witiuk K, Fergusson DA (2018). The effect of surgery report cards on improving radical prostatectomy quality: the SuRep study protocol. BMC Urol.

[15] Schlomm T, Huland H, Graefen M (2014). Improving outcome of surgical procedures is not possible without adequate quality measurement. Eur Urol.

[16] Fry S, Challacombe B (2019). Both sides of the scalpel: the patient and the surgeon view. Nat Rev Urol.

[17] Martin NE, Massey L, Stowell C, Bangma C, Briganti A, Bill-Axelson A, Blute M, Catto J, Chen RC, D’Amico AV, Feick G, Fitzpatrick JM, Frank SJ, Froehner M, Frydenberg M, Glaser A, Graefen M, Hamstra D, Kibel A, Mendenhall N, Moretti K, Ramon J, Roos I, Sandler H, Sullivan FJ, Swanson D, Tewari A, Vickers A, Wiegel T, Huland H (2015). Defining a standard set of patient-centered outcomes for men with localized prostate cancer. Eur Urol.

[18] Morgans AK, van Bommel AC, Stowell C, Abrahm JL, Basch E, Bekelman JE, Berry DL, Bossi A, Davis ID, de Reijke TM, Denis LJ, Evans SM, Fleshner NE, George DJ, Kiefert J, Lin DW, Matthew AG, McDermott R, Payne H, Roos IA, Schrag D, Steuber T, Tombal B, van Basten JP, van der Hoeven JJ, Penson DF, Advanced Prostate Cancer Working Group of the International Consortium for Health Outcomes M (2015). Development of a standardized set of patient-centered outcomes for advanced prostate cancer: an international effort for a unified approach. Eur Urol.

[19] Evans SM, Millar JL, Moore CM, Lewis JD, Huland H, Sampurno F, Connor SE, Villanti P, Litwin MS (2017). Cohort profile: the TrueNTH Global Registry—an international registry to monitor and improve localised prostate cancer health outcomes. BMJ Open.

[20] Protopapa E, van der Meulen J, Moore CM, Smith SC (2017). Patient-reported outcome (PRO) questionnaires for men who have radical surgery for prostate cancer: a conceptual review of existing instruments. BJU Int.

[21] Liedberg F, Gudjonsson S, Xu A, Bendahl PO, Davidsson T, Mansson W (2017). Long-term third-party assessment of results after continent cutaneous diversion with Lundiana pouch. BJU Int.

[22] Jenkins DP, Cooper G (2017). Publicly available outcome data for individual surgeons: lessons from cardiac surgery. Eur Urol.

[23] Khadhouri S, Miller C, Fowler S, Hounsome L, McNeill A, Adshead J, McGrath JS, Oncology BSo (2018). The British Association of Urological Surgeons (BAUS) radical prostatectomy audit 2014/2015—an update on current practice and outcomes by centre and surgeon case-volume. BJU Int.

